# Vapors of Essential Oils from Boreal Forest Plants: A Potential New Tool for Controlling Postharvest Gray Mold of Tomato Fruit

**DOI:** 10.3390/plants15192941

**Published:** 2026-09-26

**Authors:** Maxime Delisle-Houde, Marianne Piochon, Antoine Roy-Lemieux, André Pichette, Josianne Savard, Russell J. Tweddell

**Affiliations:** 1 Département de Phytologie, Université Laval, Québec, QC G1V 0A6, Canada; maxime.delisle-houde.1@ulaval.ca (M.D.-H.); antoine.roy-lemieux.1@ulaval.ca (A.R.-L.); 2 Centre de Transformation et Valorisation de Bioproduits, Université du Québec à Chicoutimi, Chicoutimi, QC G7H 2B1, Canada; mpiochon@uqac.ca (M.P.); apichett@uqac.ca (A.P.); j3savard@uqac.ca (J.S.)

**Keywords:** essential oils, *Botrytis cinerea*, Labrador tea, sweet gale, black spruce, jack pine

## Abstract

Botanical pesticides, natural pesticides that are derived from plants, and notably essential oils (EOs), represent a potentially effective, eco-friendly, and safe alternative to conventional pesticides. This study evaluated in airtight glass chambers the effect of EOs from different boreal forest plants on *Botrytis cinerea* mycelial growth and on the development of tomato gray mold, a disease caused by *B. cinerea*. EO vapors from Labrador tea, black spruce, jack pine and, to a lesser extent, from sweet gale and balsam poplar were shown at a concentration of 200 µL/L of air to significantly reduce *B. cinerea* mycelial growth. At concentrations varying from 20 to 200 µL/L of air, EO vapors from Labrador tea, black spruce, and jack pine significantly influenced *B. cinerea* growth kinetics in a dose-dependent manner. Mycelial growth of *B. cinerea* was strongly delayed in the presence of vapors of either EO at concentrations of 100, 150, and 200 µL/L of air. Exposure of tomato fruits to the vapors of tested EOs at 50 µL/L of air was shown to significantly inhibit the development of gray mold. EO from Labrador tea was particularly effective by completely inhibiting (during the exposure period) the development of the disease when applied at a concentration of 100 µL/L of air. GC-MS analyses revealed that the main compound present in the vapors produced by the EOs from black spruce, Labrador tea, and jack pine was α-pinene (42%), sabinene (59%), and α-pinene (79%), respectively. This study suggests for the first time the possibility of using EOs from boreal forest plants as botanical pesticides to repress *B. cinerea* mycelial growth and to control tomato gray mold.

## 1. Introduction

The use of conventional pesticides is increasingly raising concerns due to their potentially harmful effects on human health and the environment. Conventional pesticides pose significant risks to aquatic and terrestrial biodiversity [1], soil fertility [2], and water quality [3], and are bioaccumulated in the food chain [4]. In regard to human health, exposure to conventional pesticides is associated with respiratory disorders, skin problems, endocrine disruption, cancer, and reproduction failures [5]. In addition, the development of resistance in pests/pathogens populations to certain conventional pesticides or groups of pesticides makes these pesticides less efficient or completely inefficient [6].

The biocidal properties of plant extracts against pathogens/pests of cultivated plants are receiving increasing attention. This interest is explained by the fact that botanical pesticides—natural pesticides that are derived from plants—represent an eco-friendly alternative to the use of conventional pesticides [7,8]. Plant extracts include essential oils (EOs), a unique and complex mixture of volatile organic compounds that are non-persistent in water and soil, biodegradable with low environmental toxicity, and with rare exceptions, non-toxic to fish and mammals [9,10,11]. EOs are known to have numerous biological activities that could be exploited in agriculture for plant protection, including antifungal/anti-oomycete, antibacterial, insecticidal, nematicidal, and herbicidal activities [12,13,14,15].

Numerous studies have been conducted to investigate the potential of EOs extracted from temperate and tropical plants, such as basil (*Ocimum basilicum*), lavender (*Lavandula angustifolia*), oregano (*Origanum vulgare*), rosemary (*Rosemarinus officinalis*), anise (*Pimpinella anisum*), and thyme (*Thymus vulgaris*), to control the pathogens/pests of cultivated plants [14,16,17,18], while very few studies have investigated the potential of EOs from boreal forest plants. A recent study revealed the toxicity of EOs from Labrador tea (*Ledum groenlandicum* syn. *Rhododendron groenlandicum*), sweet gale (*Myrica gale*), black spruce (*Picea mariana*), jack pine (*Pinus banksiana*), and balsam poplar (*Populus balsamifera*) against various pathogens and pests affecting crops [15]. Sweet gale, balsam poplar, Labrador tea, jack pine, and black spruce are plant species found in the northern part of the North American continent. Black spruce and jack pine are conifers belonging to the Pinaceae family. Labrador tea and sweet gale are aromatic shrubs belonging to the Ericaceae family and Myricaceae family, respectively. Balsam poplar is a common hardwood tree of the family Salicaceae [19].

The present study further investigated the potential of these EOs to control fungal plant diseases using the tomato (*Solanum lycopersicum*)–*Botrytis cinerea* pathosystem, where the fungus *B. cinerea* causes a tomato disease called gray mold. The objectives of this study were (1) to evaluate, in vitro, the antifungal properties of vapors produced by EOs from sweet gale, balsam poplar, Labrador tea, jack pine, and black spruce against *B. cinerea*; (2) to evaluate the effect of the EO vapors showing the strongest antifungal activity on the development of tomato gray mold; and (3) to identify the main compounds present in the EO vapors showing the strongest antifungal activity using GC-MS.

## 2. Results

### 2.1. Effect of Vapors from EOs on Mycelial Growth of B. cinerea

The effects of vapors produced by EOs (200 µL/L of air) from Labrador tea, sweet gale, jack pine, balsam poplar, and black spruce on the mycelial growth of *B. cinerea* after 48 h of incubation are shown in Figure 1. EO vapors from Labrador tea, jack pine, black spruce, sweet gale, and balsam poplar significantly (*p* ≤ 0.05) reduced the mycelial growth compared to the control. The radial growth of *B. cinerea* in the presence of EOs from balsam poplar, sweet gale, jack pine, black spruce, and Labrador tea was, respectively, 6.8, 1.3, 0, 0, and 0 mm, while the growth in the absence of EO vapors (control) was 12.7 mm (Figure 1).

The growth curves of *B. cinerea* incubated in the presence of vapors generated by different concentrations of EOs from jack pine, black spruce, and Labrador tea are shown in Figure 2. The radial growth evaluated after incubation periods of 24 and 48 h was significantly reduced in the presence of vapors generated by EOs from jack pine (Figure 2a) and black spruce (Figure 2b) at concentrations of 20, 50, 100, 150, and 200 µL/L of air compared with the control. However, vapors from jack pine and black spruce EOs at concentrations below 100 µL/L of air did not significantly reduce the mycelial growth of *B. cinerea* rated after an incubation period of 72 h (Figure 2a,b) as compared with the control. Vapors from Labrador tea EO at all tested concentrations significantly reduced the radial growth after an incubation period of 24, 48, and 72 h (Figure 2c) compared with the control. At concentrations of 100, 150, and 200 µL/L of air, Labrador tea EO completely inhibited (fungistatic activity) the growth of *B. cinerea*. The growth of the fungus in an atmosphere free of vapors following a 72 h exposure to Labrador tea EO at 100, 150, or 200 µL/L of air is presented in Figure 3. The exposure was not lethal (i.e., there was no fungicidal activity) since growth was observed in an atmosphere free of vapors. However, radial growth was delayed in a dose-dependent manner by exposure to Labrador tea EO.

### 2.2. Effect of Exposure of Tomato Fruits Inoculated with B. cinerea to EO Vapors on Gray Mold Severity

The effects of vapors from black spruce, jack pine, and Labrador tea EOs on the development of gray mold on tomato fruit are presented in Figure 4. At a concentration ≥ 50 μL/L of air, vapors from the tested EOs significantly reduced the severity of the disease. At 200 μL/L of air, vapors from black spruce and jack pine EOs strongly but not completely repressed the development of gray mold (Figure 4a,b), while vapors of Labrador tea EO at concentrations of 100, 150, and 200 μL/L of air completely repressed the development of the disease (Figure 4c).

### 2.3. Headspace GC-MS Profile of Vapors Produced by EOs from Black Spruce, Labrador Tea, and Jack Pine

The volatile profiles of the EOs from black spruce, Labrador tea, and jack pine were characterized using headspace GC-MS analysis. The main compounds (relative abundance > 5%) detected in the vapor phase are presented in Figure 5. Overall, seven monoterpene hydrocarbons were consistently detected as major constituents, including α-pinene, β-pinene, camphene, sabinene, 3-carene, p-cymene, and D-limonene. In black spruce EO vapors, α-pinene (42%), camphene (27%), β-pinene (10%), and 3-carene (9%) were the predominant compounds. Labrador tea EO vapors were dominated by sabinene (59%), followed by α-pinene (19%), β-pinene (12%), and p-cymene (6%). Jack pine EO vapors were mainly composed of α-pinene (79%), β-pinene (12%), and D-limonene (5%).

## 3. Discussion

EOs are promising natural biocidal agents with potential applications in agro-industries for the management of pathogens and pests of crops. A recent study revealed for the first time the toxicity of EOs from sweet gale, balsam poplar, Labrador tea, jack pine, and black spruce against a broad range of pathogens and pests of cultivated plants [15]. The study investigated the antifungal/prophylactic activity of these EOs against *B. cinerea*, a destructive pathogen causing serious losses in more than 200 crop species worldwide [20].

EO vapors from Labrador tea, black spruce, jack pine, and, to a lesser extent, sweet gale, and balsam poplar at a concentration of 200 µL/L of air were shown to significantly reduce *B. cinerea* in vitro mycelial growth after 48 h of incubation. The effects of EOs from Labrador tea, black spruce, and jack pine at different concentrations on *B. cinerea* growth kinetics were then evaluated. At concentrations varying from 20 to 200 µL/L of air, they were shown to significantly influence *B. cinerea* growth kinetics in a dose-dependent manner. Mycelial growth of *B. cinerea* was strongly delayed in the presence of vapors of either EO at concentrations of 100, 150 and 200 µL/L of air. The effect of EO vapors from Labrador tea was particularly severe. Indeed, EO vapors from Labrador tea at 100, 150, and 200 µL/L of air completely inhibited the mycelial growth of *B. cinerea*; the exposure was, however, not lethal. This shows that Labrador tea EO vapors over a given concentration have a fungistatic activity against *B. cinerea*. Previous studies have reported the antifungal activity of EOs from oregano, lavender, rosemary, black caraway (*Nigella sativa*), peppermint (*Mentha* × *piperita*), lemongrass (*Cymbopogon citratus*), Shirazi thyme (*Zataria multiflora*), clove (*Syzygium aromaticum*), luisa herb (*Aloysia citriodora*), citronella (*Cymbopogon nardus*), and cinnamon (*Cinnamomum verum*) against *B. cinerea* [21,22,23,24]. The present study reports, for the first time, the antifungal activity of EOs from Labrador tea, black spruce, and jack pine against *B. cinerea*.

*Botrytis cinerea* typically infects tomato fruits in the fields and greenhouses through wounds causing a disease called gray mold. Gray mold is considered one of the most destructive postharvest tomato diseases in both field and greenhouse productions [25], where the humidity and temperature conditions are conducive to fungal development [25,26,27]. The disease is characterized by the soft decay of fruit tissues accompanied by the growth of fuzzy gray-brown mold. The application of phytosanitary products in the field before storage is recommended to prevent the disease [28]. However, phytosanitary products may be leached, and spray restrictions could reduce the effectiveness of this strategy. In Canada, several active ingredients are registered for use in fields and greenhouses against gray mold. However, only one product, hydrogen peroxide, is registered to control warehouse rot in tomato [29]. The vapors from Labrador tea, black spruce, and jack pine EOs were investigated herein for their effect on the development of *B. cinerea* gray mold on postharvest tomato fruit. The exposure of tomato fruits to the vapors of tested EOs at 50 µL/L of air was shown to significantly inhibit the development of the disease. Labrador tea EO was particularly effective, completely inhibiting the development of the disease when applied at a concentration of 100 µL/L of air. Previous studies have reported the potential of EOs extracted from temperate, subtropical, or tropical plants for the control of gray mold on postharvest tomato fruit; EOs extracted from oregano [30,31], peppermint [32], *Aloysia polystachya* [33], *Torreya grandis*, *Eriobotrya japonica*, *Citrus medica* [34], *Tetraclinis articulata* [35], and *Cupressus sempervirens* [36] were shown to be efficient in controlling the disease. The present study reports for the first time the efficacy of EOs extracted from boreal forest plants for the control of postharvest tomato gray mold.

Headspace GC-MS analyses were performed with EOs from Labrador tea, black spruce, and jack pine in order to identify the main volatile compounds released in the atmosphere to which *B. cinerea* and *B. cinerea*-inoculated tomato fruits were exposed. The main compounds detected in the vapor phase of jack pine EO were α-pinene, β-pinene, and D-limonene. α-pinene and β-pinene have previously been reported among the major constituents of jack pine EO [37,38]. However, in a comprehensive study analyzing leaf terpenes from 420 jack pine trees, Lapp and von Rudloff [37] also identified limonene, myrcene, car-3-ene, β-phellandrene, and bornyl acetate as major constituents of the oil of this species. The main compounds detected in the vapor phase of black spruce EO were α-pinene, camphene, β-pinene, and 3-carene. α-pinene and camphene [38,39,40], as well as β-pinene [41,42], have also been identified among the predominant compounds in black spruce EO. Our results are in agreement with those of Francezon and Stevanovic [41], who reported α-pinene as the major constituent of this EO. Although several studies have reported bornyl acetate as the major constituent of black spruce EO [38,39,42], the latter was not detected herein. This study is the first to report 3-carene among the major constituents of black spruce EO. The main compounds detected in the vapors of Labrador tea EO were sabinene, α-pinene, β-pinene, and p-cymene. Sabinene was previously reported as a main compound of different samples of Labrador tea EO [38,43,44]. α-pinene and β-pinene were also previously reported as main compounds in samples of Labrador tea EO [43]. However, limonene and β-selinene, which are considered as the most important compounds of the EO extracted from Labrador tea aerial parts [43], were not detected herein at relative contents exceeding 5%. p-cymene was previously reported as a minor compound in Labrador tea EO [43]. The chemical profile of plant extracts such as EOs can vary due to several factors, including the geographic location of the plant material, the timing of the harvest (phenological cycle), the extraction method employed, and the type of analysis used (e.g., HPLC, UPLC, GC-MS, GC-FID) [43,45].

Sabinene, α-pinene, β-pinene, camphene, 3-carene, p-cymene, or limonene were previously shown to have antifungal activity against different species of the genera *Candida* (α-pinene, β-pinene, 3-carene, camphene, limonene, D-limonene, sabinene), *Aspergillus* (3-carene, sabinene), *Trichophyton* (sabinene, limonene), and *Fusarium* (p-cymene) [46,47,48,49,50,51,52,53]. This suggests that *B. cinerea* mycelial growth inhibition could be attributable, at least in part, to α-pinene, β-pinene, camphene, and/or 3-carene (black spruce), to α-pinene, β-pinene, and/or D-limonene (jack pine), and to sabinene, α-pinene, β-pinene, and/or p-cymene (Labrador tea). Similarly, the repressive effects of the EOs on gray mold development could result from the direct toxicity of the aforementioned monoterpenes on *B. cinerea* present on the fruit. However, it cannot be discounted that the least abundant compounds could have some antifungal activity [54]. Finally, considering that α-pinene has previously been reported to induce plant defense mechanisms in *Cassia occidentalis* [55], it is possible that the prophylactic effect of the EOs was, at least in part, achieved through the stimulation of natural plant defenses.

## 4. Conclusions

In the current context, where the world demand for food is expected to continually increase and where the population is increasingly concerned about the environment, eco-friendly alternatives to conventional pesticides are urgently needed. Botanical pesticides based on EOs are promising alternatives to conventional pesticides. The volatility of EOs make them useful as possible fumigants for stored commodity protection. The present study documents, for the first time, the antifungal activity of EOs from Labrador tea, black spruce, and jack pine against *B. cinerea*, as well as their repressive effects on the development of postharvest gray mold on tomato fruit, highlighting their potential for disease management and for reducing postharvest losses.

However, further research is needed to assess the vapor-phase stability of each EO, elucidate the antifungal mode of action of their individual compounds, and identify those responsible for the observed inhibitory effects. Once these mechanisms have been clarified, EOs from Labrador tea, black spruce, and jack pine will have to be evaluated under commercial postharvest conditions (in vivo) with both artificially and naturally inoculated tomato fruits to determine their potential for integration into postharvest disease management strategies for tomato. Such studies will also help determine whether the experimental concentrations tested in the present study are realistic and applicable under commercial storage conditions, and assess the costs and feasibility of scaling up these treatments for commercial applications. Because postharvest treatments must maintain fruit quality, future research will have to assess their effects on shelf life, fruit color, firmness, and flavor. Finally, the dose, formulation, effects on non-target organisms (i.e., other pathogens and beneficial microbiota), environmental persistence, and worker exposure will have to be thoroughly evaluated prior to potential registration for commercial use.

## 5. Materials and Methods

### 5.1. Essential Oils (EOs)

EOs from Labrador tea, sweet gale, black spruce, jack pine, and balsam poplar were provided by SiliCycle Inc. (Québec, QC, Canada). EOs were extracted from the aerial parts of the plants using steam distillation, as previously described by Roy-Lemieux et al. [15].

### 5.2. Botrytis cinerea

*Botrytis cinerea* [virulent strain *Bc*-LEDP; GenBank accession no. PV590312 (ITS)] was provided by the Laboratoire d’expertise et de diagnostic en phytoprotection [ministère de l’Agriculture, des Pêcheries et de l’Alimentation (MAPAQ), Québec, QC, Canada]. The fungus was grown on potato dextrose agar (PDA; Becton, Dickinson and Company, Sparks, MD, USA) at room temperature (22.5 ± 1 °C). Fungal suspension was prepared as described in Arabzadeh et al. [56]. Five 5 mm PDA plugs covered with actively growing mycelium of *B. cinerea* were cultivated over 7 days at room temperature under agitation (150 rpm) in a flask containing 100 mL of potato dextrose broth (Becton, Dickinson and Company). Mycelium was recovered using cheesecloth and then homogenized with a blender in sterile physiological water (0.5% NaCl). The fungal suspension was then adjusted to a concentration of 1 × 10^5^ propagules/mL using a hemocytometer (Hausser Scientific, Horsham, PA, USA).

### 5.3. Effect of Vapors from EOs on Mycelial Growth of B. cinerea

PDA plugs (10 mm diameter) covered with actively growing mycelium of *B. cinerea* were placed on PDA in Petri plates. Each Petri plate (without a lid) containing one PDA plug was subsequently placed in an airtight glass chamber (empty internal volume = 500 cm^3^) containing a sterile filter paper (area of 38.48 cm^2^) soaked—or not (control)—with 100 µL of the EOs of either balsam poplar, Labrador tea, sweet gale, black spruce, or jack pine, corresponding to 200 µL of EO/L of air. Glass chambers were then incubated in darkness at room temperature. Radial growth of the fungus was determined using a ruler after 48 h of incubation. The experiment was conducted as a completely randomized design with six replicates, with an airtight glass chamber containing a Petri plate being the experimental unit.

The effects of the vapors produced by different quantities of EOs from Labrador tea, jack pine, and black spruce on *B. cinerea* mycelial growth were also evaluated. The experiment was conducted as previously described, except that each sterile filter paper was soaked with either 0 (control), 10, 25, 50, 75, or 100 µL of EO, corresponding to 0, 20, 50, 100, 150 or 200 µL of EO/L of air. The radial growth of the fungus was determined after 24, 48, and 72 h of incubation (darkness, room temperature). Petri plates containing a mycelial plug that showed no growth after 72 h were transferred to a glass chamber free of EO vapors. After different periods (24, 48, and 72 h) of incubation (darkness, room temperature), radial growth was determined. The experiments were conducted as a completely randomized design with six replicates, with an airtight glass chamber containing a Petri plate being the experimental unit. Experiments with each EO were conducted separately.

### 5.4. Effect of Exposure of Tomato Fruits Inoculated with B. cinerea to EO Vapors on Gray Mold Severity

Tomato (Micro Tom variety) fruits were collected from plants cultivated in a greenhouse environment, as described in Barrada et al. [57]. They were surface-sterilized using a 5% sodium hypochlorite solution (for 3 min) followed by a 70% ethanol solution (for 3 min), as described in Conti et al. [58], and then rinsed twice with distilled sterile water. Four wounds were made on each fruit using a sterile needle. Fruits were then soaked for 2 min in *B. cinerea* suspension (1 × 10^5^ propagules/mL) and subsequently placed in an airtight glass chamber (empty internal volume = 500 mL) containing a sterile filter paper that was previously soaked with 0 (control), 10, 25, 50, 75, or 100 µL of EOs from either Labrador tea, jack pine, or black spruce, corresponding to 0, 20, 50, 100, 150, or 200 µL of EO/L of air. The glass chambers were then incubated in darkness at room temperature. After 96 h of incubation, the disease severity was determined according to the following 0–4 scale: 0 (no symptoms); 1 (fruit surface covered by *B. cinerea* mycelium ≤ 10%); 2 (10% < fruit surface covered by *B. cinerea* mycelium ≤ 25%); 3 (25% < fruit surface covered by *B. cinerea* mycelium ≤ 50%); and 4 (fruit surface covered by *B. cinerea* mycelium > 50%). The experiments were conducted via a completely randomized design with nine replicates, with an airtight glass chamber containing two fruits being the experimental unit. Experiments with each EO were conducted separately.

### 5.5. Headspace GC-MS Analysis

Headspace chromatographic analyses of the EOs were performed on an Agilent 8890 gas chromatography system equipped with a PAL RSI 85 automatic headspace autosampler (Agilent Technologies, Inc., Santa Clara, CA, USA). The GC was coupled to a 5977B GC/MSD mass spectrometer (Agilent Technologies). Glass vials (30 mL, sealed with certified screw caps with PTFE silicone septa; Agilent Technologies) containing the samples (25 μL) were equilibrated for 10 min under agitation (250 rpm) at 30 °C. The GC injection port was set at 250 °C, and the injection volume was 0.2 mL in a split mode (ratio: 200:1). The GC system was equipped with a HP-5MS capillary column (30 m × 0.25 i.d. mm × 0.25 µm film thickness; Agilent Technologies). The GC oven temperature was programmed to 50 °C for 2 min, then ramped to 175 °C at 3 °C/min, and then subsequently ramped to 250 °C at 10 °C/min, with a final hold time of 10 min. Helium was used as the carrier gas, and the flow rate was 0.8 mL/min. The MSD transfer line, MS source, and MS quadrupole temperatures were set at 280, 230, and 150 °C, respectively. The ionization was carried out in the electron impact (EI) mode at 70 eV, and the MS data were acquired in full scan monitoring mode from *m*/*z* 45–300. Data were analyzed using Agilent MassHunter Qualitative Analysis software (version 10.0). Compounds were initially tentatively identified by comparison of their mass spectra with those contained in the NIST MS Search 2.3 database. The identities of the main compounds were then confirmed by comparison of both the mass spectra and retention times with authentic analytical standards analyzed under identical chromatographic conditions. Volatile compounds were reported as relative abundances expressed as percentage peak areas (% area) based on GC-MS total ion chromatogram peak area normalization, and were used for comparative purposes to describe differences in volatile composition of the EOs.

### 5.6. Statistical Analysis

All statistical analyses were performed in R (version 4.1.1; R Core Team, 2021, Vienna, Austria). Analysis of variance (ANOVA; car package) and the non-parametric Kruskal–Wallis test were performed to assess the effects of EO vapors on the mycelial growth of *B. cinerea*. The assumptions of parametric tests were evaluated using Levene’s test (car package) for homogeneity of variances and the Shapiro–Wilk test for normality. When assumptions were not met, rank transformation was applied and non-parametric analyses were also performed. As previously reported by Delisle-Houde and Tweddell [59], rank-based analyses provide a robust alternative when parametric assumptions are violated. Treatment effects were analyzed using post hoc LSD tests for mycelial growth (agricolae package). Given that two fruits were placed within the same glass chamber in the experiment to assess the effect of exposing tomato fruits inoculated with *B. cinerea* to EO vapors on gray mold severity, a linear mixed-effects model (lme4 package) was used. Treatment effects were analyzed using a type II analysis of variance for gray mold severity (car package), followed by pairwise comparisons of estimated marginal means with Tukey adjustment (emmeans package).

## Figures and Tables

**Figure 1:**
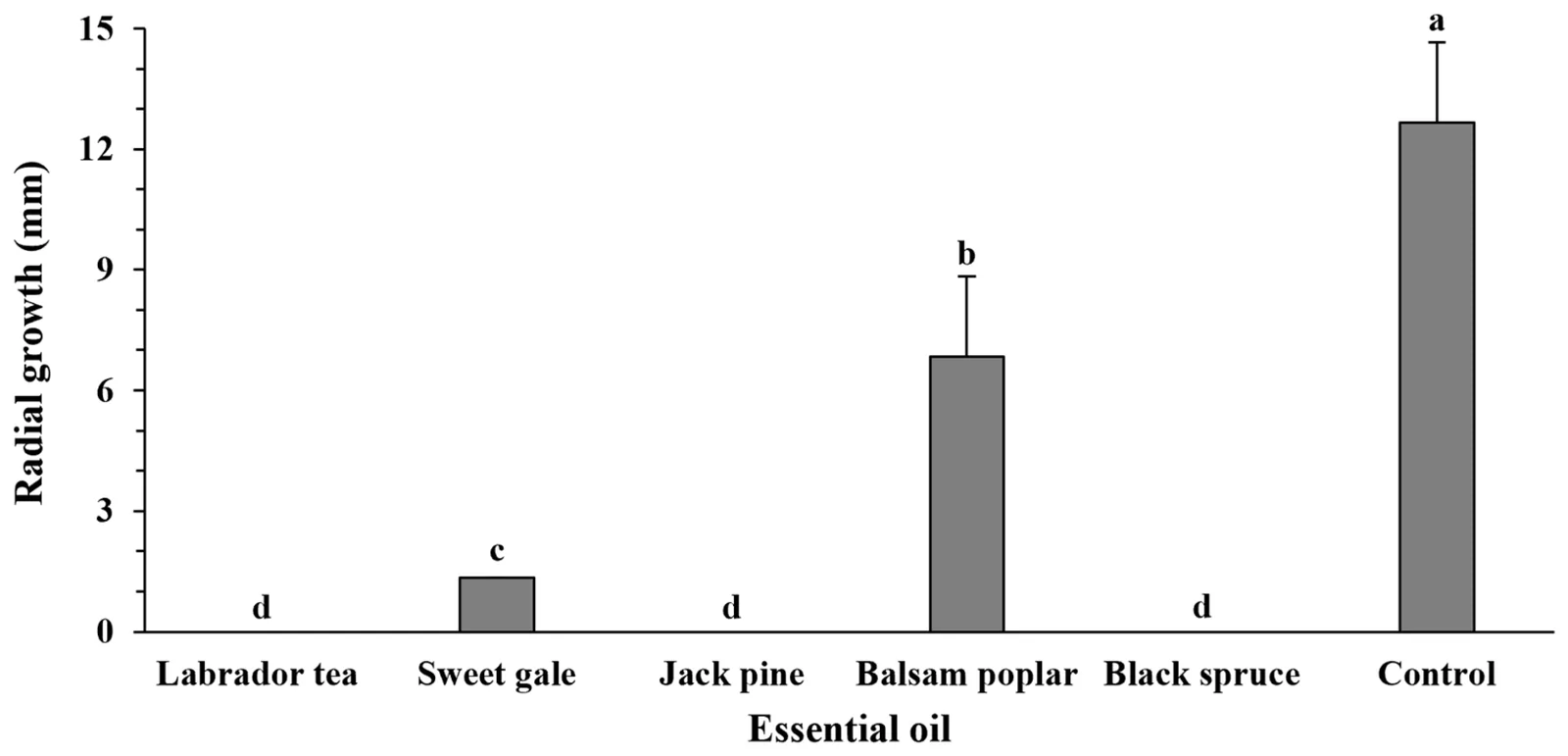
Radial growth of *Botrytis cinerea* in the absence (control) and presence of the vapors of different essential oils (200 μL/L of air) after an incubation period of 48 h. Each value represents the mean of six replicates ± standard error. Means with the same letter are not significantly different according to a post hoc LSD test (*p* ≤ 0.05).

**Figure 2:**
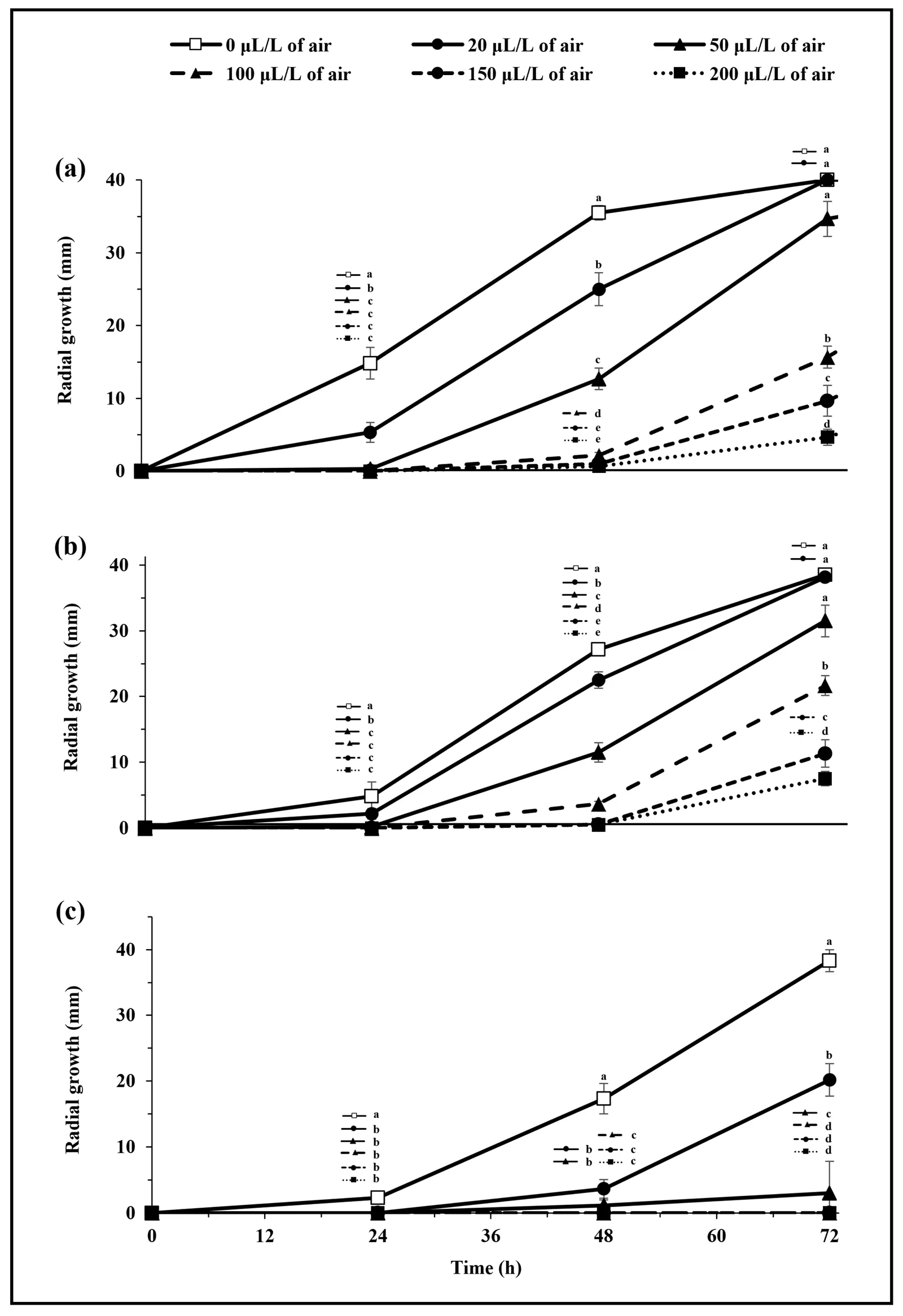
Radial growth of *Botrytis cinerea* over time in the presence of vapors of essential oils from jack pine (**a**), black spruce (**b**), and Labrador tea (**c**) at different concentrations [0 (control), 20, 50, 100, 150, and 200 μL/L of air]. Each value represents the mean of six replicates ± standard error. For each incubation time, means with the same letter are not significantly different according to post hoc LSD test (*p* ≤ 0.05).

**Figure 3:**
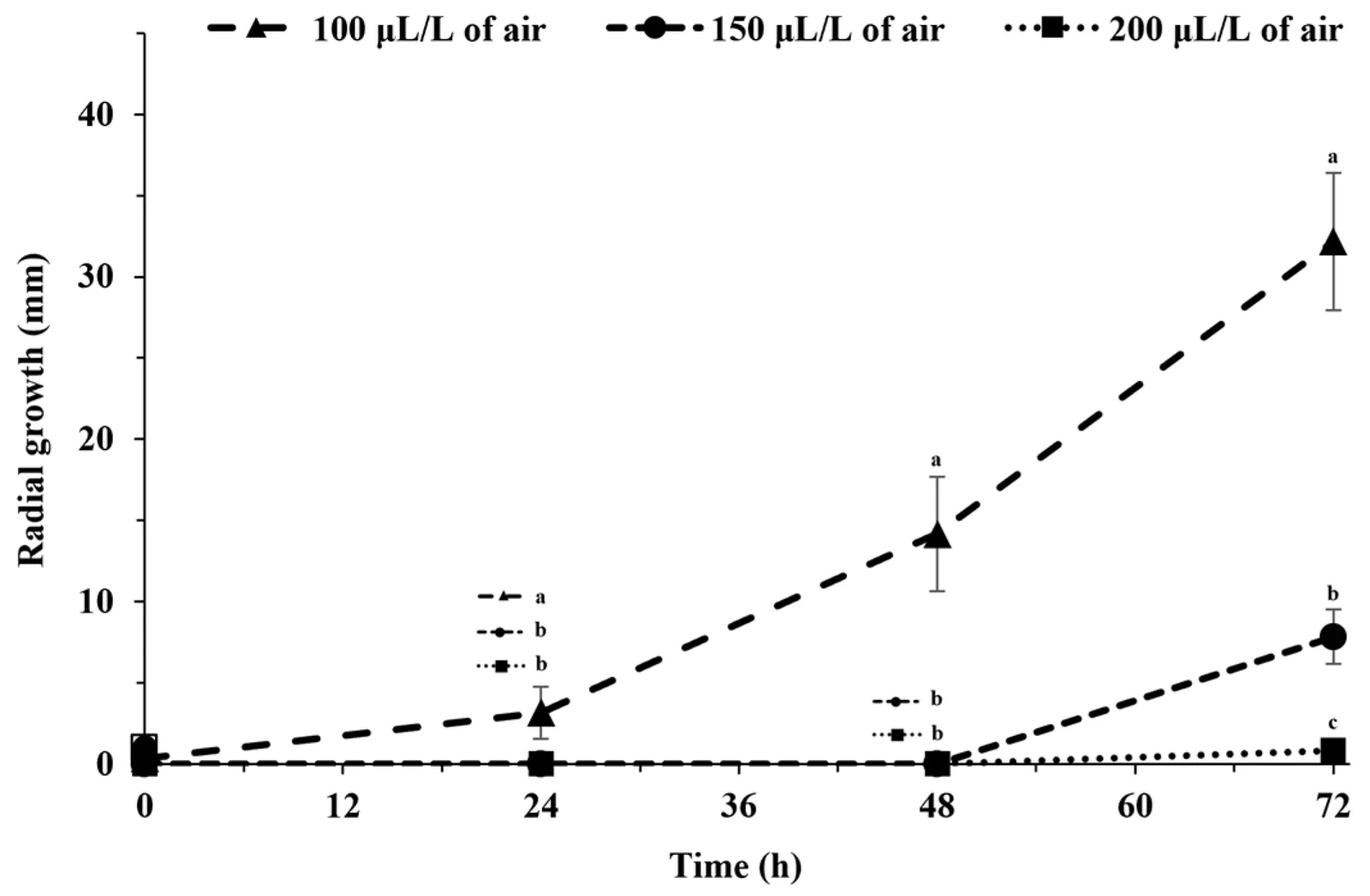
Radial growth of *Botrytis cinerea* over time in an atmosphere free of vapors following a 72 h exposure to vapors of Labrador tea essential oil at concentrations of 100, 150 or 200 μL/L of air. Each value represents the mean of six replicates ± standard error. For each incubation time, means with the same letter are not significantly different according to post hoc LSD test (*p* ≤ 0.05).

**Figure 4:**
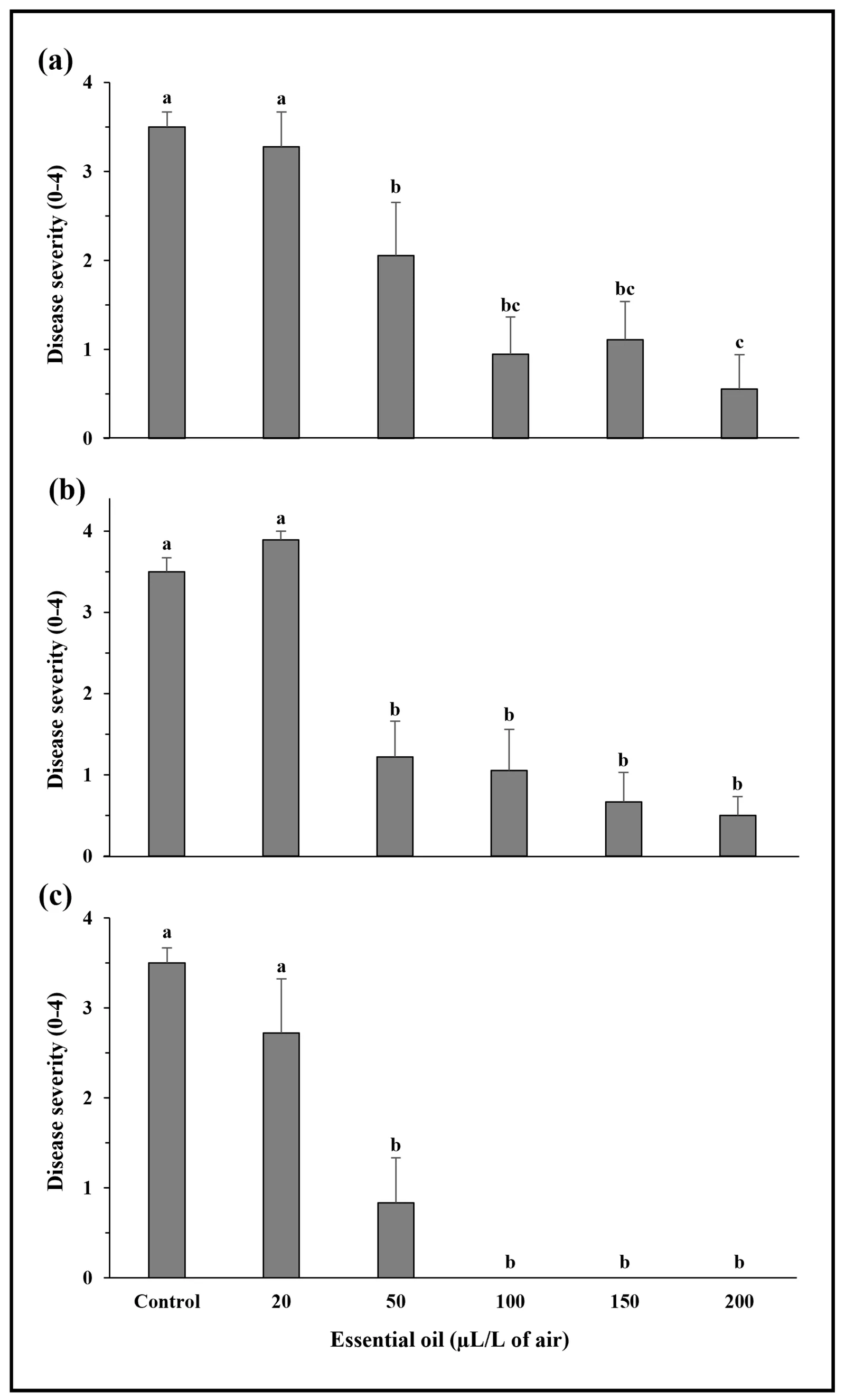
Effect of exposure of tomato fruits inoculated with *Botrytis cinerea* to vapors of essential oils from black spruce (**a**), jack pine (**b**) and Labrador tea (**c**) at different concentrations [0 (control), 20, 50, 100, 150, and 200 μL/L of air] on gray mold severity. Each value represents the mean of nine replicates ± standard error. Means with the same letter are not significantly different according to post hoc pairwise multiple comparisons with Tukey’s adjustment (*p* < 0.05).

**Figure 5:**
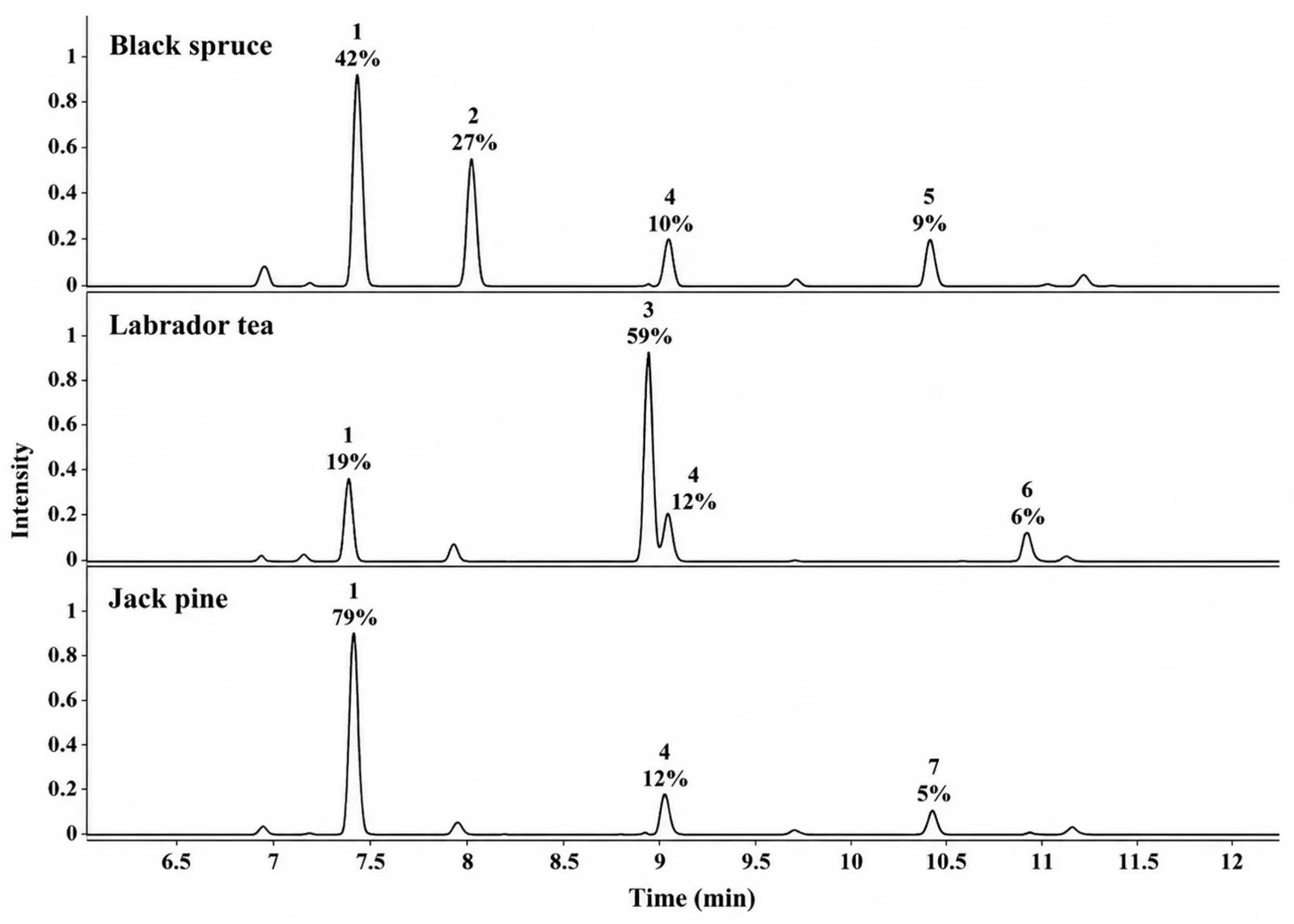
Identification and relative area (%) of the main compounds present in the essential oil vapors from black spruce, Labrador tea, and jack pine. Only compounds representing more than 5% of each essential oil are listed [1: α-pinene; 2: camphene; 3: sabinene; 4: β-pinene; 5: 3-carene; 6: p-cymene; 7: D-limonene].

## Data Availability

Rough data can be requested from the corresponding author.
